# Review of Recent Advances in Cold-Sprayed Coatings for Accident-Tolerant Fuel Cladding

**DOI:** 10.3390/ma19061056

**Published:** 2026-03-10

**Authors:** Yuqi Mou, Yunjie Zhou, Hong Zhou, Rui Yang, Jing Huang, Ye Tian, Shuangjie Wu, Ping Zhou, Meiqi Song, Jin Han, Hua Li

**Affiliations:** 1 Department of Materials Science & Engineering, Zhejiang University of Technology, Hangzhou 310014, China; mouyuqi@nimte.ac.cn (Y.M.); hanau@163.com (J.H.); 2 State Key Laboratory of Advanced Marine Materials, Ningbo Institute of Materials Technology and Engineering, Chinese Academy of Sciences, Ningbo 315201, China; tianye@nimte.ac.cn (Y.T.); wushuangjie@nimte.ac.cn (S.W.); zhouping@nimte.ac.cn (P.Z.); songmeiqi@nimte.ac.cn (M.S.); 3 Zhejiang Engineering Research Center for Biomedical Materials, Zhejiang-Japan Joint Laboratory for Antibacterial and Antifouling Technology, Ningbo Institute of Materials Technology and Engineering, Chinese Academy of Sciences, Ningbo 315201, China; 4 State Grid Shanghai Municipal Electric Power Company, Shanghai 200122, China; zyj13601620501@163.com (Y.Z.); sd58zhou501@163.com (H.Z.)

**Keywords:** cold spray technology, accident-tolerant fuel, nuclear energy, cold-sprayed coatings, chromium, FeCrAl alloys, MAX phase composites

## Abstract

The 2011 Fukushima accident highlighted the vulnerability of traditional Zr alloy fuel cladding under loss-of-coolant accident (LOCA) conditions, prompting the development of accident-tolerant fuel (ATF) systems. A promising near-term solution involves depositing protective coatings on existing Zr alloy cladding. Among various deposition techniques, cold spray technology has emerged as one of the leading methods due to its solid-state, low-temperature process, which minimises thermal degradation and allows for the deposition of a wide range of high-performance materials. This review provides a comprehensive examination of recent advances in cold-sprayed coatings for ATF cladding, beginning with an overview of the fundamentals of cold spray technology and its specific advantages for nuclear applications. The core of the review critically analyses three primary coating systems: Cr, FeCrAl alloys, and MAX phase composites, with a particular focus on Cr coatings, as they have been more extensively studied compared to the other two material systems. Key coating properties, including microstructure of the coating-substrate interface, mechanical properties, thermal conductivity, oxidation resistance, irradiation tolerance, and performance under normal operation and simulated LOCA conditions, are discussed in detail, with particular emphasis on the potential of cold-sprayed Cr coatings to enhance Zr alloy cladding. Cr coatings demonstrate significant improvements in oxidation resistance and irradiation stability, but also face challenges such as high-temperature interfacial reactions. To address these issues, promising solutions, such as diffusion-barrier bilayer systems, are being explored. Additionally, the review discusses FeCrAl and MAX phase composite coatings, highlighting their promising long-term performance under extreme conditions. The review concludes with recommendations for further research to optimise cold spray processes and ensure the robustness of coatings in operational reactor environments.

## 1. Introduction

Nuclear power offers several advantages, including high efficiency, stability, and low carbon emissions. In recent years, technological advancements and the increasing global demand for clean energy have further elevated the importance of nuclear power within the global energy mix [1]. According to the International Energy Agency, nuclear power accounted for 9.1% of global electricity generation in 2023 [2]. Among nuclear reactors worldwide, light water reactors (LWRs), which include pressurised water reactors (PWRs) and boiling water reactors (BWRs), make up the vast majority, accounting for approximately 71% and 14% of the total, respectively [3]. The nuclear fuel rods used in these reactors primarily consist of low-enriched UO_2_ fuel pellets and Zr alloy cladding [4].

As cladding materials for nuclear fuel, Zr alloys are favoured for their high thermal conductivity, excellent corrosion resistance, and relatively low neutron absorption cross-section [5]. Furthermore, Zr alloys exhibit good mechanical strength and stability under neutron irradiation and at high temperatures [6], thereby maintaining the structural integrity of fuel rods during regular reactor operation. However, the loss-of-coolant accident (LOCA) at the Fukushima Daiichi Nuclear Power Plant in 2011, triggered by the combined effects of an earthquake and tsunami, highlighted significant issues with Zr alloys under LOCA conditions [6]. During LOCA, the inadequate cooling causes the temperature of the fuel rods to rise rapidly, leading to a rapid reaction between the Zr alloys and high-temperature steam. This reaction produces considerable heat and hydrogen, which can result in explosions and catastrophic releases of radioactive materials [7]. In response to the risks posed by LOCA scenarios, the U.S. Department of Energy introduced the concept of accident-tolerant fuels (ATFs) in 2012. The primary objectives of ATFs include enhancing the thermal conductivity of the fuel to reduce its temperature and slowing the corrosion and oxidation processes between the cladding material and steam at high temperatures [8]. Thus, the development of ATFs has focused on improving fuel properties, enhancing cladding characteristics, and optimising the reaction kinetics between the fuel and steam [8,9].

Rather than developing entirely new cladding materials, a more feasible short- to medium-term solution is to deposit protective coatings onto existing Zr alloy cladding. Various coating material systems have shown promise in providing ATF-compliant nuclear fuel rods. These include Cr coatings [10,11,12], FeCrAl coatings [11,13,14,15,16], and MAX phase composite coatings [17,18,19]. The primary techniques used for fabricating these coatings include physical vapour deposition (PVD) [20,21], laser additive manufacturing [22,23], and plasma spraying [24,25]. Cold spray technology has emerged in recent years as a promising approach for producing nuclear fuel cladding coatings, owing to its unique advantages [26], including the ability to produce dense, strong coatings without high temperatures, preventing oxidation and thermal degradation. It also minimizes thermal damage, enhances oxidation and corrosion resistance, and allows for the deposition of a wide range of materials, making it a versatile and effective method for improving cladding performance under extreme conditions.

This paper presents an overview of the fundamental principles of cold spray technology, reviews the development of cold-sprayed accident-tolerant nuclear fuel cladding coatings by materials, and evaluates their key application-related properties. The review critically examines three primary coating systems, Cr, FeCrAl alloys, and MAX phase composites, with a particular focus on Cr coatings due to their more extensive research compared to the other two material systems. The review delves into key coating properties such as microstructure at the coating-substrate interface, mechanical properties, thermal conductivity, oxidation resistance, irradiation tolerance, and performance under both normal reactor operation and simulated LOCA conditions. Additionally, FeCrAl and MAX phase composite coatings are also highlighted for their promising long-term performance under extreme conditions, providing potential alternatives for future nuclear fuel cladding systems. The review concludes by outlining future research directions to further optimise cold spray processes and ensure the robustness of these coatings in real-world reactor environments, ultimately contributing to the development of safer and more efficient accident-tolerant fuels.

## 2. Basics of Cold Spray

### 2.1. Cold Spray Systems

Cold spray is a process that utilises high-speed gas to accelerate solid material particles to supersonic velocities, which are then directly deposited onto a substrate surface to form a coating [27]. The cold spray process can be classified into two categories based on the propulsive gas pressure [28]: low-pressure cold spray (<1 MPa) and high-pressure cold spray (>1 MPa).

**Figure 1:**
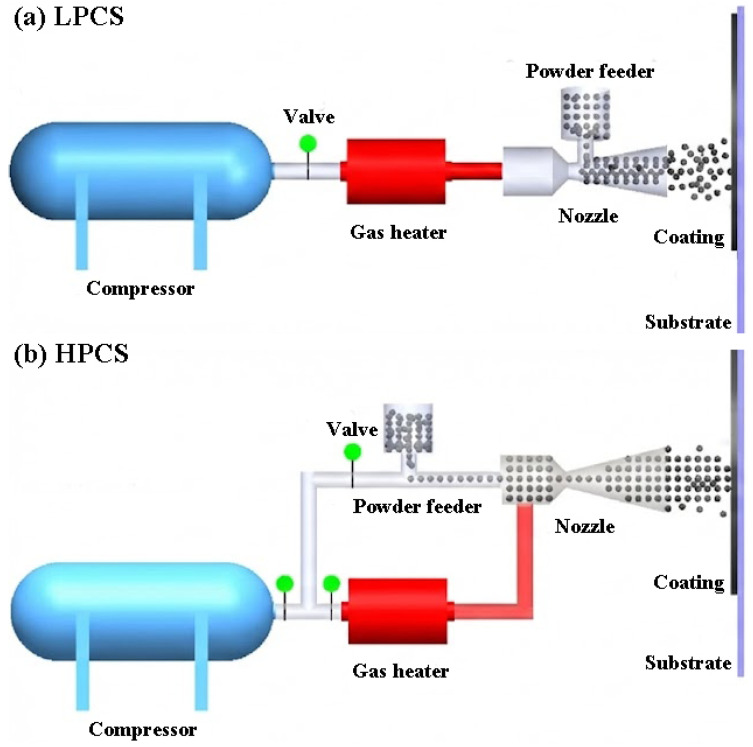
Schematic of (**a**) low-pressure and (**b**) high-pressure cold spray systems [29].

The low-pressure cold spray system generally utilises a portable air compressor with a simplified nozzle design, in which the powder is injected into the divergent section of the nozzle (Figure 1a). These design features contribute to the flexibility of the system and result in significantly lower equipment and processing costs compared to high-pressure systems. However, due to the lower particle velocity in low-pressure systems, their applicability is limited to relatively soft materials with low melting points, such as Cu and Al.

In contrast, the high-pressure cold spray system uses compressed gases such as nitrogen or helium to accelerate the powder to supersonic velocity. As illustrated in Figure 1b, the compressed gas is split into two separate streams: the propulsive gas, which passes through a gas heater to reach a high temperature, and the carrier gas, which flows through a powder feeder to mix with feedstock particles. These two gas streams are then combined before entering a de-Laval nozzle, where they expand and form a supersonic flow of gas and particles. The high-velocity particles impact the substrate, forming a coating at a temperature well below their melting point. Due to higher particle velocities, high-pressure cold spray systems can deposit a broader range of materials. Given that the materials used for ATF cladding coatings exhibit high mechanical strength and high melting temperatures, high-pressure cold spray is the only suitable method for their deposition. Hence, the term ‘cold spray’ refers specifically to ‘high-pressure cold spray’ in this review.

### 2.2. Bonding Mechanisms and Theory

The interfacial bonding mechanisms of cold-sprayed coatings are strongly influenced by the dynamic interplay between process parameters and the material system. Experimental studies have shown that factors such as the propellant gas (pressure and temperature), nozzle configuration, particle impact velocity, powder feed rate, and substrate surface topography collectively govern the deformation behaviour and bonding mode of the particles [30,31]. By modulating the impact kinetic energy, localised strain rate, and interfacial temperature rise, these parameters work together to influence the bonding mechanisms during the cold spray process.

Currently, based on experimental observations, particle–substrate bonding is primarily achieved through a combination of mechanisms, including jetting [32,33], mechanical interlocking [34,35], localised melting [35,36], oxide-layer fracturing [37,38], and metallurgical bonding [38,39,40]. These mechanisms work synergistically to form a strong and stable bonded interface. Although a fully unified theory of the deposition mechanism of cold-sprayed coatings has not yet been established, the adiabatic shear instability theory has emerged as the dominant framework for explaining bonding behaviour in this process, due to its broad applicability and experimental verifiability [41,42,43,44]. According to this theory, under extreme strain rates, intense plastic deformation generates an adiabatic temperature rise, leading to thermal softening that overrides strain hardening and ultimately triggering localised shear instability.

### 2.3. Advantages of Cold Spray for Depositing ATF Cladding Coatings

When considering the development of ATF cladding coatings, various deposition techniques have been explored, each with its own set of advantages and limitations. These methods include PVD, laser additive manufacturing, and plasma spraying, all of which offer unique benefits but also face significant challenges when applied to nuclear fuel cladding. A careful examination of these techniques helps to better understand the need for alternative methods, such as cold spray, which provide distinct advantages under the demanding conditions of nuclear reactor environments.

The advantage of PVD lies in its ability to produce a dense coating with a high surface finish, and its properties can be precisely tailored through multilayer structural design [20,21]. However, PVD requires a high vacuum, which limits its production efficiency, particularly for slender fuel tubes [45]. Laser additive manufacturing enables the formation of a strong metallurgical bond between the coating and substrate and the low porosity in the coating [22,23]. However, the high thermal input can cause grain coarsening and phase transformations in the Zr substrate, leading to the formation of a brittle heat-affected zone. Additionally, controlling the dilution rate of the substrate is challenging, which may result in deviations from the intended coating composition. Plasma spraying offers several advantages, including low cost and high efficiency, a wide range of sprayable materials, good mechanical properties, and the ability to minimize substrate deformation [46]. However, coatings produced by this method often exhibit a layered, porous structure with oxide inclusions [24,25]. In the LOCA conditions, high-temperature steam can penetrate the interconnected pore and directly oxidize the zirconium matrix, causing coating failure. Furthermore, oxidation of the powder during plasma spraying can reduce both the thermal conductivity and mechanical properties of the coating.

Cold spray offers several distinct advantages for depositing ATF cladding coatings. One of the key benefits is its ability to produce dense, strong coatings without the need for high temperatures, thereby preventing oxidation and thermal degradation of sensitive coating materials [47]. Cold spray also avoids thermal damage and distortion of materials that can occur at elevated temperatures during deposition. As a result, the deposited coatings are expected to significantly enhance the oxidation and corrosion resistance, as well as the structural integrity of the fuel cladding, ensuring stability in high-temperature and irradiated environments. Furthermore, cold spray allows for the deposition of a wide range of materials, including those with high melting points, such as Cr metal, FeCrAl alloys, and MAX phase composites, all of which show great potential for improving cladding performance under extreme conditions [26]. Overall, these advantages position cold spray as an effective and versatile method for developing robust ATF cladding coatings. Table 1 summarises the key properties of the coatings by various deposition techniques.

### 2.4. Cold Spray Coating Deposition on Cylindrical Surfaces

Currently, the transition of cold spray techniques from laboratory samples to the industrial production of full-scale fuel rods (approximately 4 m in length and 9.5 mm in diameter) continues to face several engineering challenges. To achieve a uniform coating on the surface of slender cylindrical components, a helical scanning path is commonly employed, where the workpiece rotates at high speed while the spray gun moves axially. For cladding tubes with a diameter of only 9.5 mm, the particle beam generated by the nozzle (typically following a Gaussian distribution) not only covers the tube wall area perpendicular to the nozzle but also impacts areas at non-perpendicular angles. When particles strike the tube wall at such angles, deposition efficiency significantly decreases, and porosity increases. To ensure good overall coating quality, precise control over the spraying distance and overlap rate is essential [48]. The inherent surface roughness of cold-sprayed coatings (approximately 5~10 μm in Ra) can severely affect the thermal-hydraulic performance of reactors, such as altering the critical heat flux. Consequently, post-processing (grinding/polishing) is vital to achieve a sufficiently smooth surface finish. This imposes stringent requirements on coating thickness uniformity, as the grinding process removes peaks, and excessive variations in thickness may lead to the localised exposure of the substrate [49].

## 3. Cr Coatings

Cr shows significant potential for ATF applications due to several favourable properties, including its coefficient of thermal expansion, which is similar to that of the Zr substrate, as well as its thermophysical properties that closely match those of Zr. Additionally, Cr can accommodate coordinated deformation with the substrate during temperature increases and can form a protective CrO_2_ layer under high-temperature conditions [50]. To date, numerous studies have successfully deposited cold-sprayed Cr coatings on Zr substrates, demonstrating excellent resistance to high-temperature oxidation.

### 3.1. Effect of Processing Parameters on the Cold-Sprayed Cr Coatings

#### 3.1.1. Shape of Particle

Maier et al. investigated the feasibility of preparing Cr coatings on zirconium alloy cladding surfaces using cold spraying and compared the effects of different powder shapes on the coatings [51]. The gas-atomised powder, which exhibited a spherical morphology, demonstrated significant plastic deformation and a tendency for dynamic recrystallisation during high-velocity impact. The resulting coatings contained a high density of sub-micron grains and severely deformed regions, indicating that the deposition mechanism was dominated by adiabatic shear and intense plastic flow. In contrast, electrolytic Cr powder, characterised by its irregular shape, showed less pronounced deformation in the coatings. The bonding mechanism in this case relied more on mechanical interlocking and surface roughness than on plastic deformation. Due to its insufficient ductility and limited deformation capacity, the cold spray process failed to induce widespread adiabatic shear instability or dynamic recrystallisation effectively. As a result, the improvement in hardness was limited. This highlighted that the physical morphology and preparation process of the powder directly influence its deformation behaviour during cold spraying and the final bonding characteristics of the coating.

#### 3.1.2. Feedstock Annealing

Yeom et al. investigated the enhancement of deposition efficiency in cold-sprayed Cr coatings on Zr-alloy substrates by annealing electrolytic Cr powder [52]. The as-received powder was annealed at 800 °C for 5 h in an argon atmosphere, resulting in significant microstructural changes: the original fine, irregular grains transformed into well-defined equiaxed grains. As a consequence, the nano-hardness decreased due to strain relief and recrystallisation. Using the annealed powder, the deposition efficiency increased dramatically, reaching up to 3 times that of the as-received powder. The resulting coatings exhibited dense, continuous microstructures with localised bands of heavily deformed grains at particle-particle interfaces, indicating improved plastic deformation upon impact. Furthermore, substrate deformation and interfacial roughness were reduced compared to coatings produced from the as-received powder. High-temperature oxidation tests demonstrated excellent performance: after exposure to 1200 °C for 20 min in air, a thin chromium oxide layer (~4.5 μm) formed on the coating surface, effectively protecting the underlying Zr alloy.

#### 3.1.3. Propulsive Gas

Maier et al. and Yeom et al. also compared the effects of using nitrogen versus a nitrogen–helium mixture as the propellant medium for Cr coating deposition [51,52]. Due to the lower molecular weight of helium, its inclusion in the mixture significantly increased the gas flow velocity and particle impact speed, thereby enhancing particle kinetic energy and plastic deformation capability. This promoted the formation of a denser coating structure with stronger bonding. In contrast, using pure nitrogen as the propellant resulted in lower particle velocities, which limited coating densification and interfacial bond strength. Although this study did not directly compare the mechanical properties of the two coating types, it clarified, from a process-mechanism perspective, that using gas mixtures could optimise deposition outcomes by increasing particle velocity. This approach is particularly advantageous for coating systems that rely on high-impact energy to achieve strong bonding.

**Figure 2:**
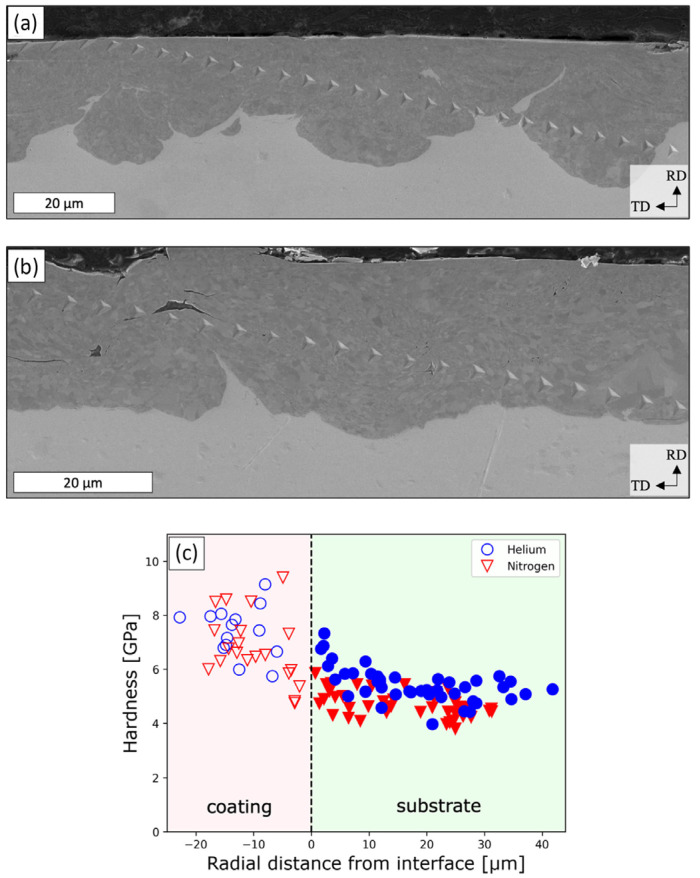
Nanoindentation tests on the coatings: (**a**) indentation sites of the helium-propelled coating, (**b**) indentation sites of the nitrogen-propelled coatings, and (**c**) the hardness results [53].

Alamkoizidis et al. systematically investigated the influence of propellant gases (helium and nitrogen) on the microstructure and mechanical properties of Cr coatings prepared via cold spraying on zirconium alloy cladding tubes [53]. The study demonstrated that nitrogen-propelled coatings exhibited higher porosity and a smoother coating/substrate interface, along with significantly lower residual compressive stress compared to helium-propelled coatings (Figure 2). Due to higher porosity and lower bonding strength, nitrogen-propelled coatings exhibited cracking initiated at a lower axial strain, and the crack density increased more rapidly in the low strain range. However, at higher strains, both coatings eventually reached a similar saturated crack density and exhibited similar crack toughening mechanisms, such as bridging, deflection, and blunting. Additionally, the higher particle velocity achieved with helium propellant in helium-propelled coatings resulted in greater plastic deformation and increased interfacial roughness, leading to a more non-uniform coating thickness distribution. In contrast, nitrogen-propelled coatings, processed at higher preheating temperatures and pressures, promoted more significant grain refinement. The study concluded that while nitrogen offered lower cost and produced more uniform coatings, its higher porosity could compromise the oxidation and corrosion resistance of the coating under reactor operating conditions. Therefore, future performance evaluations in high-temperature, corrosive, and irradiated in-reactor environments are essential.

### 3.2. Properties of the Cold-Sprayed Cr Coatings

#### 3.2.1. Microstructure of the Coating–Substrate Interface

**Figure 3:**
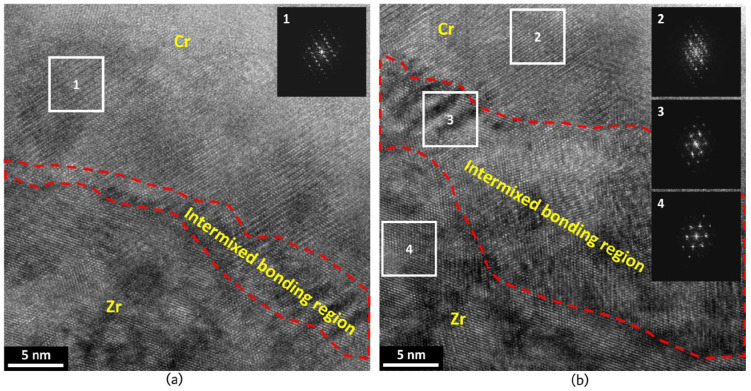
HRTEM images of the cold-sprayed Cr-coating/Optimised ZIRLO™ interface, along with the corresponding Fast Fourier transforms (FFTs) of (**a**) the Cr coating, and (**b**) the Cr coating, Zr-alloy substrate, and the intermixed bonding region [12].

**Figure 4:**
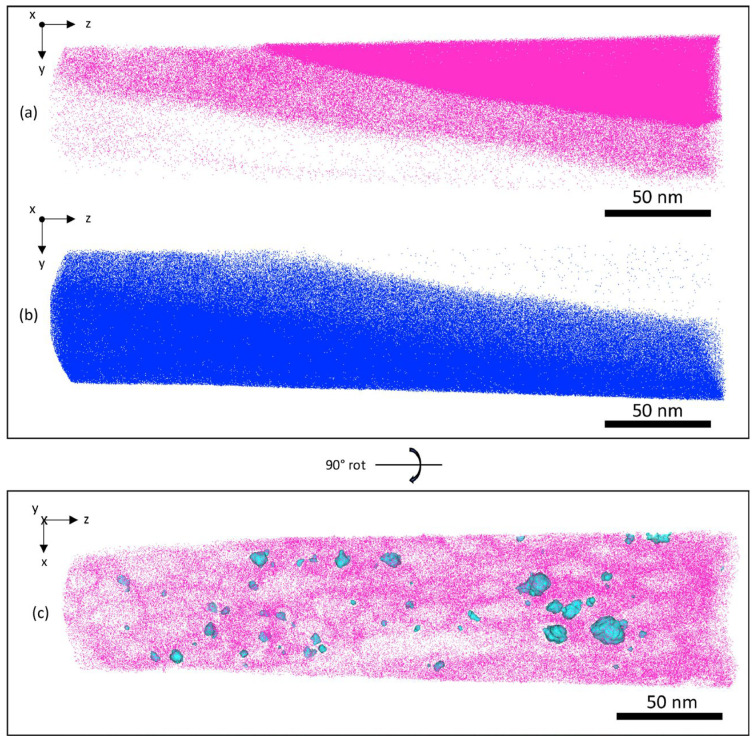
3D reconstruction of APT data from the cold-sprayed Cr-coating/Optimised ZIRLO™ interface and the surrounding area, showing the distribution of (**a**) Cr atoms in pink, (**b**) Zr atoms in blue (both representing 30% of ions), and (**c**) a 15 nm slice of the intermixed bonding region (the light-blue particles (isosurfaces at 4 at.% C) indicate areas of high carbon concentration) [12].

Fazi et al. used high-resolution Transmission Electron Microscopy (HRTEM) and Atom Probe Tomography (APT) to analyse the coating-substrate interface of cold-sprayed Cr coating on Zr alloy cladding [12]. The results revealed an intermixed bonding region approximately 10–20 nm thick (Figure 3), where Cr and Zr had diffused into each other (Figure 4). This mixed zone primarily consisted of 60–70 at.% Zr and 30–40 at.% Cr, with trace amounts of oxygen and alloying elements such as Fe, Sn, and Nb from the substrate.

The high-strain-rate deformation induced during the cold spray process enhanced Cr diffusion into the Zr substrate, forming a distorted hexagonal close-packed structure within the zone (Figure 3). This interface is crucial to the mechanical properties of the coating, as it ensures both strong mechanical interlocking and crystallographic coherency between the coating and the substrate [12].

The dynamic recrystallisation induced by the cold-spray process also affected the Zr substrate, forming a nanocrystalline layer approximately 1–2 μm thick. This nanocrystalline layer was characterised by fine grains and a high density of grain boundaries, which further facilitated the diffusion of Cr along the grain boundaries into the Zr substrate [12,54].

#### 3.2.2. Mechanical Properties

**Figure 5:**
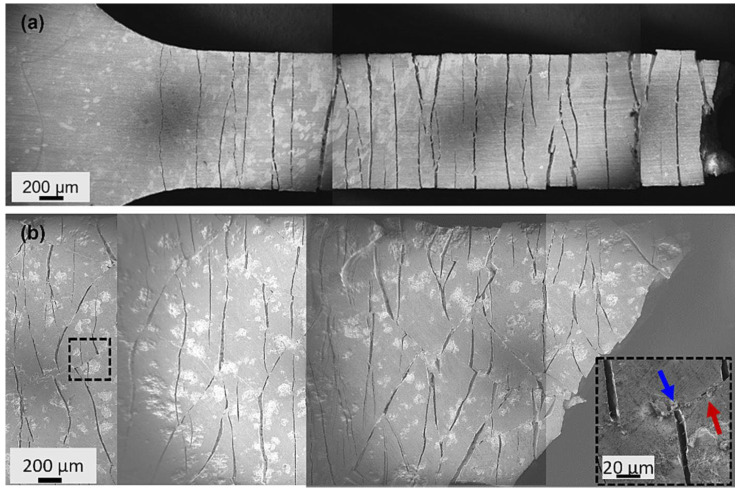
SEM images showing cracking along the surface of (**a**) the as-deposited Cr coating and (**b**) the annealed Cr coating, with red and blue arrows indicating the cracking in the axial and transverse directions, respectively [55].

Dabney et al. used in situ synchrotron diffraction tensile tests to gain detailed insights into the deformation and failure mechanisms of the cold-sprayed Cr/Zr alloy system [55]. The study revealed that the as-deposited coating exhibited a high dislocation density, with a significantly greater proportion of screw dislocations compared to edge dislocations. This characteristic deviated from the typical deformation behaviour of BCC metals under traditional low strain rates and could be attributed to the exceptionally high strain rates during cold spraying, along with the localised adiabatic temperature rise induced by particle impact. Under tensile loading, the coating displayed brittle cracking at approximately 1% strain. According to the scanning electron microscopy (SEM) image (Figure 5a), the cracks propagated perpendicular to the loading direction, but no coating delamination was observed, indicating strong bonding at the coating/substrate interface. After annealing, the dislocation density in the coating decreased by roughly one order of magnitude, the proportion of edge dislocations further reduced, and the microstructure became more homogeneous. Additionally, the crack propagation paths became more tortuous (Figure 5b), demonstrating enhanced resistance to crack propagation and macroscopic toughness. Notably, the Cr coating did not significantly affect the tensile properties of the Zr alloy substrate. Its yield strength, ultimate tensile strength, and elongation remained comparable to those of the uncoated substrate, indicating good mechanical compatibility between the coating and the substrate.

In addition, pretreatment of the feedstock powder can also influence the mechanical properties of the coating. As mentioned in the study by Yeom et al. [52], subjecting electrolytic Cr powder to an appropriate annealing treatment prior to deposition could not only significantly improve deposition efficiency but also indirectly enhanced the mechanical properties and interfacial bonding quality of the coating by optimising the deformability and microstructure of the Cr powder.

#### 3.2.3. Thermal Conductivity

Thermal conductivity is a critical property for materials used in ATF cladding, as it directly influences heat transfer performance and nuclear reactor safety. In the context of ATF cladding, maintaining effective heat transfer is essential to prevent excessive temperatures that could compromise the structural integrity of the cladding material, especially under accident conditions such as LOCA.

Jo et al. investigated the critical heat flux of Cr-coated Zr alloy substrates for ATF cladding, focusing on coatings applied via cold spraying [56]. The study indicated that the Cr-coated surfaces did not significantly affect critical heat flux compared to uncoated Zircaloy-4, as post-coating polishing treatment maintained surface characteristics similar to those of commercial light water reactor cladding. Although the Cr-coated surfaces were more hydrophobic, this difference in wettability did not notably alter critical heat flux. The results indicated that surface finish and material properties played a more significant role in critical heat flux than the coating method itself.

Lee et al. examined the heat transfer properties of cold-sprayed Cr coatings on zirconium-alloy substrates under flow boiling conditions [57]. The study found that the cold-sprayed Cr coating exhibited the highest surface roughness, leading to increased microcavities that promoted bubble nucleation. This resulted in a 10.9% increase in void fraction and a 5.2% improvement in the heat transfer coefficient. However, the cold-sprayed Cr coating also reduced critical heat flux by 11.6% due to lower wettability, hindering rewetting and leading to premature vapour film formation. This balance of improved heat transfer and reduced critical heat flux underscores the importance of carefully optimising coating techniques to achieve the desired performance for accident-tolerant fuel cladding materials. Despite this, the rougher surface accelerated cooling during the post-critical heat flux quenching phase, thereby preventing surface oxidation by enhancing heat transfer.

#### 3.2.4. Oxidation Resistance

In the context of ATF cladding coating development, cold-sprayed Cr coatings have garnered significant attention for their exceptional oxidation resistance. Several studies highlight the potential of Cr coatings in improving the performance of Zr alloy cladding under high-temperature conditions, particularly in steam environments associated with LOCA. The protective mechanism of Cr coatings primarily relies on the formation of a dense, stable Cr_2_O_3_ oxide layer that acts as a barrier, preventing oxygen diffusion into the underlying substrate.

Research by Park et al. demonstrated that the cold-sprayed Cr coatings could significantly suppress oxidation in Zr alloys when exposed to high-temperature steam at 1200 °C for 3000 s [58]. In simulated LOCA conditions, Cr-coated cladding showed a remarkable improvement, including higher burst temperatures, reduced circumferential strain, and smaller rupture openings compared to uncoated Zr alloys.

**Figure 6:**
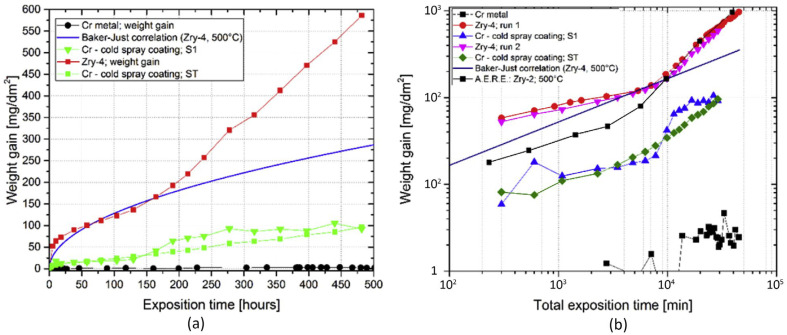
Oxidation kinetics of Zr alloy, Cr-coated samples with (ST) and without (S1) defects, and pure Cr sample at 500 °C steam: (**a**) the increase in weight over time within the oxidation chamber for Zry-4, Cr-coated samples, and pure chromium. A shift in oxidation behaviour is observed in the Zircaloy-4 samples after approximately 150 h; (**b**) comparison of the oxidation kinetics of the tested samples at 500 °C in steam with the B-J correlation and the oxidation behaviour of Zircaloy-2 [59].

The long-term efficacy of cold-sprayed Cr coatings was validated by Ševeček et al., who subjected the coatings to extended exposure in 500 °C steam environments for up to 20 days [59]. The Cr-coated samples exhibited substantially lower weight gain than uncoated Zr alloys, demonstrating the ability of the coating to resist corrosion even under prolonged exposure (Figure 6). Additionally, multiple cycles of steam oxidation at 1200 °C revealed that the Cr coatings maintained structural integrity, forming a thin protective oxide layer that shielded the substrate from further oxidation.

Studies by Maier et al. have also shown that cold spraying, particularly with atomised Cr powder, can produce coatings with excellent oxidation resistance at temperatures up to 1300 °C in air and in high-pressure steam environments [51,60]. The coatings were found to effectively inhibit the oxidation of Zr alloys, with minimal interdiffusion between the Cr and Zr during high-temperature exposure. The high-density, tightly bonded Cr_2_O_3_ layer formed on the coating surface contributed to its resilience under extreme conditions, ensuring prolonged protection of the substrate.

As mentioned in Section 3.1.2, a study by Yeom et al. further explored the potential of cold-sprayed Cr coatings by investigating the effect of annealing electrolytic Cr powder [52]. In addition to improving deposition efficiency and interfacial bonding, coatings produced with annealed electrolytic Cr powder exhibited oxidation resistance comparable to that of coatings made with gas-atomised Cr powder. The study concluded that annealed electrolytic Cr powder was a cost-effective and practical alternative to gas-atomised powder, improving deposition efficiency while maintaining coating performance. This approach significantly expands the range of feedstocks suitable for cold spray applications.

Cold-sprayed Cr coatings provide an effective solution for enhancing the oxidation resistance of Zr alloys used in ATF cladding. These coatings offer significant improvements in performance under both high-temperature air and steam conditions, with good adhesion to the substrate and minimal degradation even after extended exposure. Further research should focus on refining the cold spray process, optimising coating uniformity, and investigating long-term behaviour in irradiation environments to fully assess the potential of Cr coatings in operational reactors.

#### 3.2.5. Irradiation Resistance

In addition to oxidation resistance, cold-sprayed Cr coatings also exhibit promising irradiation resistance for ATF cladding applications. A key feature of these coatings is their dense microstructure, which arises from severe plastic deformation during cold spraying. This microstructure has been shown to delay the onset and growth of irradiation-induced defects compared to bulk Cr coatings, further enhancing their potential for use in nuclear fuel applications.

Cold-sprayed coatings initially exhibit high compressive stress, which helps prevent cracking during the early stages of reactor operation. However, as neutron fluence increases, irradiation creep can lead to stress relaxation. Ideally, the creep rate of the coating should match that of the Zr alloy matrix to prevent excessive interfacial shear stress and delamination during cladding dimensional changes caused by fuel swelling. Studies have demonstrated that cold-sprayed Cr coatings possess good irradiation creep resistance, potentially providing additional structural support for the cladding [61].

The as-deposited cold-sprayed Cr coatings exhibit a fine-grained structure with a high dislocation density, primarily due to the high strain rates during particle impact in the cold spray process. These characteristics are significant for their radiation resistance. Maier et al. conducted in situ TEM characterisation of bulk Cr and cold-sprayed Cr coatings [62]. The results showed that the irradiation with high-energy Kr ions led to the formation of dislocation loops and other defects. However, the cold-sprayed Cr coatings exhibited a much lower defect density and smaller loop sizes than bulk Cr under the same irradiation conditions. This suggests that deformation-induced defects in the cold-sprayed Cr coatings act as sinks for radiation-induced point defects, thereby mitigating the growth of larger defects.

Similar findings were reported by Dabney et al., showing that at low doses (0.1–0.7 dpa), irradiation-induced defects were less prevalent in cold-sprayed Cr coatings than in bulk Cr [63]. At higher doses (3 dpa), both materials showed increased defect formation, but the cold-sprayed Cr maintained a lower defect density and smaller loop sizes. This was attributed to pre-existing dislocation networks and grain boundaries, which effectively absorb radiation-induced defects.

The interface between the cold-sprayed Cr coating and the Zr alloy substrate also plays a critical role in the overall irradiation resistance. Dabney et al. found that high-energy ion irradiation led to the formation of a thin (~20 nm) amorphous layer at the Cr/Zr interface, which was attributed to radiation-induced thermodynamic effects [63]. The amorphisation of the interface was observed to occur with Fe dissolution and the stabilisation of Cr in a disordered state. This amorphous layer did not negatively impact the adhesion between the Cr coating and the Zr-alloy substrate, further suggesting the robustness of cold-sprayed Cr coatings under irradiation.

Recrystallisation induced by irradiation can occur in the cold-sprayed Cr coatings. According to Dabney et al., the interparticle boundaries were replaced by recrystallised grains, which contributed to the densification of the coating [63]. These structural changes, coupled with nanoscale voids, enhanced resistance to radiation-induced degradation. Nanoindentation testing of the irradiated Cr coatings showed a slight softening compared to the unirradiated state, consistent with the effects of recrystallisation and grain growth under irradiation.

The reactor environment combines high temperature, intense radiation, corrosion, and mechanical stress. Neutron irradiation can accelerate the corrosion of Zr alloys. Although Cr coatings generally exhibit excellent corrosion resistance, radiolysis of the water chemistry environment under irradiation generates oxidising species that may accelerate the dissolution of the Cr oxide film. However, the results from pool-side inspections at commercial reactors have confirmed that after several years of high neutron irradiation and normal operating coolant flow, the cold-sprayed Cr coating still remained virtually intact [64].

In summary, the above studies suggest that cold-sprayed Cr coatings exhibit strong resistance to irradiation damage, primarily due to their microstructural features, including fine grain size, high dislocation density, and defect sinks. These characteristics significantly delay the onset and growth of radiation-induced defects, making cold-sprayed Cr coatings a promising candidate for accident-tolerant fuel cladding in nuclear reactors.

### 3.3. Behaviours in Normal Operation and Simulated LOCA Conditions

#### 3.3.1. Normal Operation

**Figure 7:**
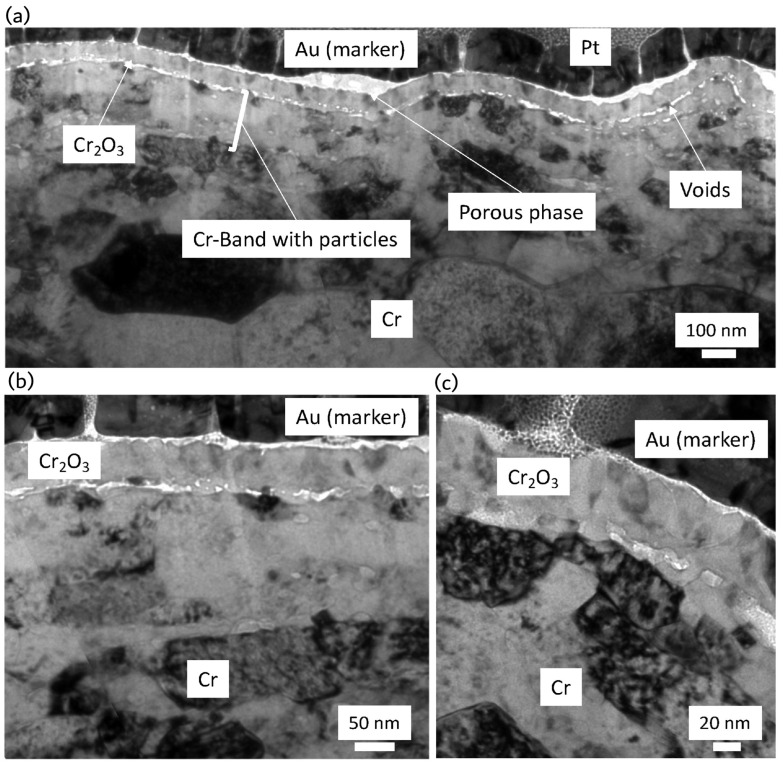
Bright field TEM images of the oxide layer developed on the external surface of the cold-sprayed Cr coating after autoclave exposure in (**a**) low, (**b**) medium, and (**c**) high magnifications [54].

Under simulated PWR environments, the Cr coating demonstrates excellent performance in preventing substrate oxidation. The study by Fazi et al. showed that, after 90 days of autoclave corrosion testing, a dense, adherent Cr_2_O_3_ oxide layer formed on the surface of the cold-sprayed Cr coating [54]. This oxide layer, approximately 80–100 nm thick (Figure 7), effectively blocked oxygen diffusion into the underlying substrate, preventing the rapid oxidation that can occur with uncoated zirconium alloys, which typically form much thicker ZrO_2_ layers under similar conditions.

**Figure 8:**
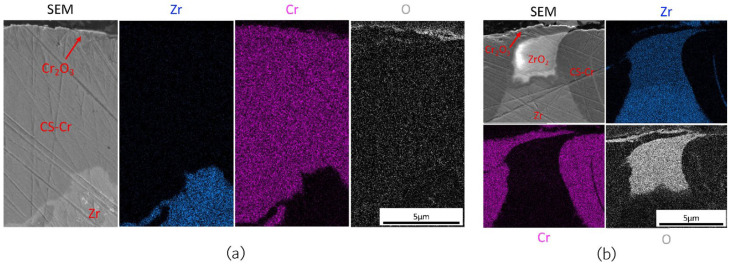
EDS mapping of the cross-section of cold-sprayed Cr coating on the Optimised ZIRLO™ cladding over (**a**) a fully coated region and (**b**) the region where a tiny section of the Zr substrate was exposed to the autoclave environment [54].

Even in regions where the Cr coating experienced localised thinning or defects, oxidation was still confined to the exposed areas, and no significant coating spallation was observed (Figure 8). This behaviour demonstrates the defect tolerance of the Cr coating, a critical feature for practical applications where minor damage can be expected. The ability of the Cr coating to maintain oxidation resistance even when compromised enhances its potential for long-term deployment in nuclear reactors.

**Figure 9:**
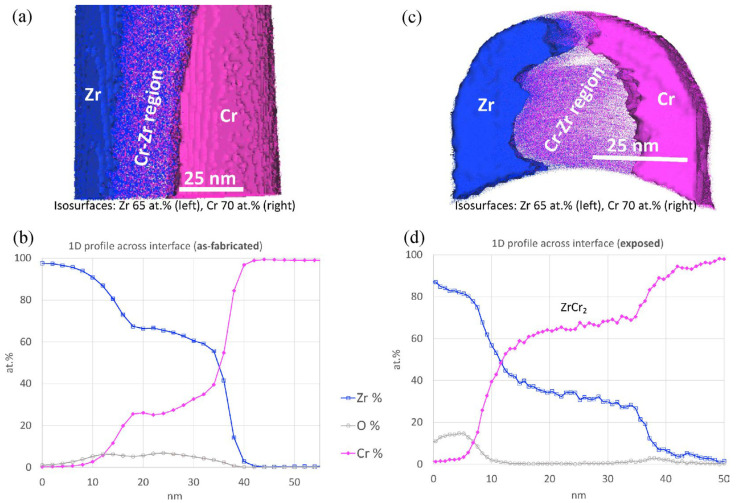
APT results at the interface of cold-sprayed Cr-coating and Zr alloy cladding, showing the 3D reconstruction of (**a**) as-fabricated and (**c**) autoclave-exposed samples, as well as (**b**,**d**) the corresponding 1D composition profile across the interface [54].

Thermal exposure over long periods can lead to gradual microstructural changes at the interface between the cold-sprayed Cr coating and the Zr alloy substrate. The Cr coating remains stable under normal operating conditions, but slow microstructural evolution occurs due to thermodynamic conditions. Precipitates of ZrCr_2_ were observed within the mixed zone at the Cr/Zr interface after long-term exposure (Figure 9) [12,54]. These small precipitates (less than 50 nm in size) suggest that the conditions for nucleation of the ZrCr_2_ phase are satisfied at operating temperatures, although its growth was limited due to the slow bulk diffusion of Zr.

**Figure 10:**
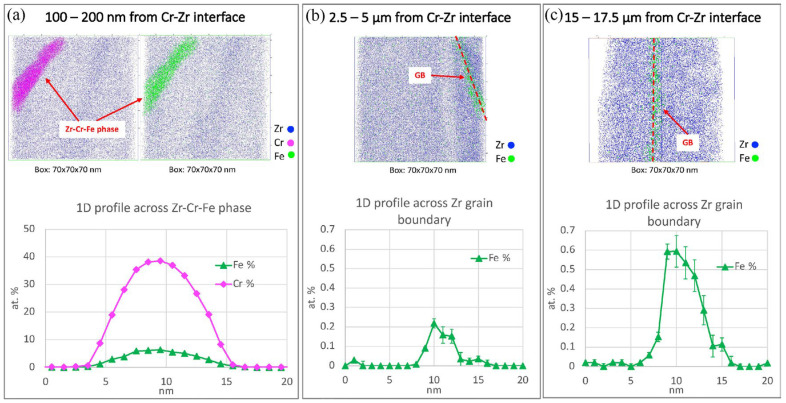
3D reconstruction of APT data at the grain boundaries extracted from the autoclave-exposed Zr-substrate at different distances from the Cr/Zr interface associated with the corresponding 1D concentration profiles across the grain boundary [54].

In addition to the formation of ZrCr_2_, Zr-Cr-Fe precipitates were detected at the grain boundaries near the interface, possibly attributed to Cr diffusion and Fe redistribution during thermal exposure (Figure 10). However, the overall microstructure of the Cr coating and the nanocrystalline Zr layer remained relatively stable during thermal exposure. Only minor grain coarsening was observed, with no large-scale recrystallisation, indicating that the coating system maintains good thermal stability under operational conditions.

#### 3.3.2. Simulated LOCA Conditions

**Figure 11:**
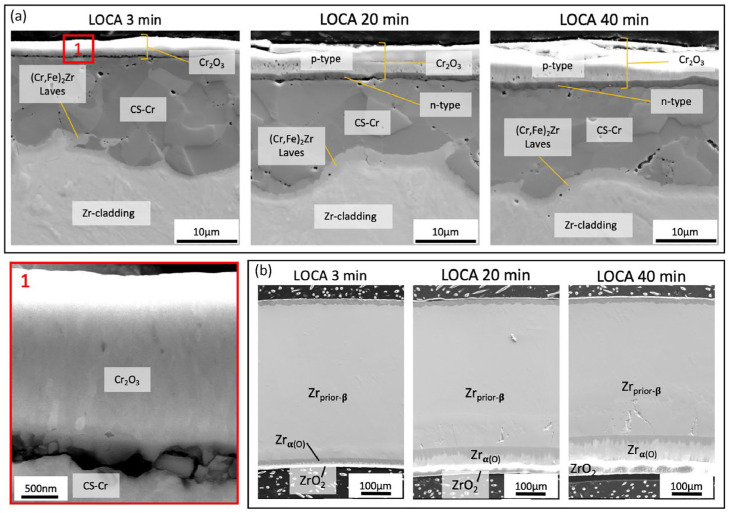
SEM images of the cross-section of the cold-sprayed Cr-coated Optimised ZIRLO™ cladding exposed to simulated LOCA conditions for 3 min, 20 min, and 40 min, respectively: (**a**) close-up of the outer cladding wall with a Cr-coating after exposure, where the contrast between n-type and p-type chromia is due to their differing electronic properties and Box 1 provides an enlarged view of the oxide layer and the oxide/metal interface after 3 min of exposure; (**b**) general view of the cladding cross-section: the uncoated inner wall of the cladding is shown, highlighting the extent of oxidation experienced by the bare Zr-alloy [65].

**Figure 12:**
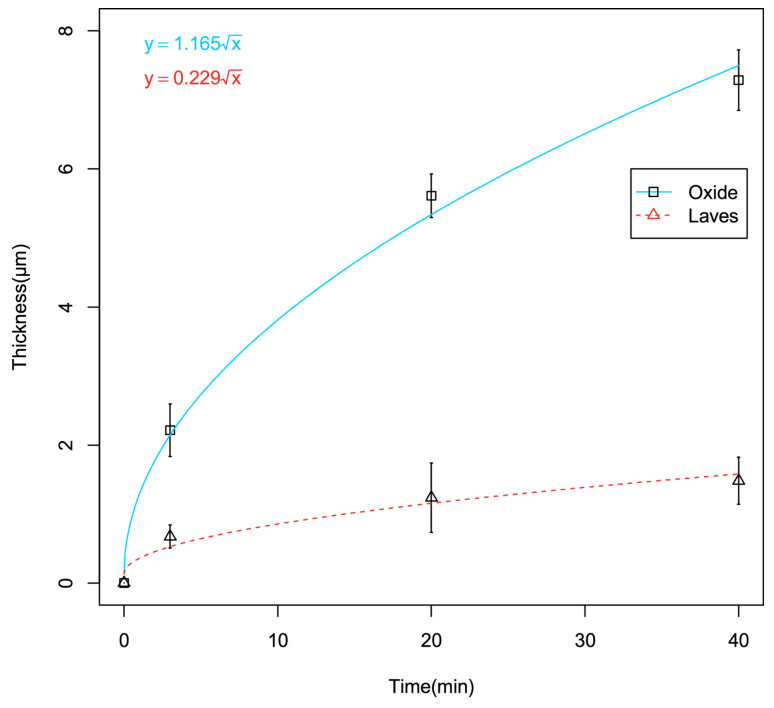
Measured thicknesses of the Cr_2_O_3_ layer and the (Cr,Fe)_2_Zr Laves phase layer as a function of simulated LOCA exposure [65].

As mentioned in Section 3.2.4, cold-sprayed Cr coatings have been widely proposed as near-term accident-tolerant enhancements for Zr-based cladding because Cr can form a slow-growing Cr_2_O_3_ layer that can restrict oxygen transport at high temperatures. Under LOCA-relevant steam exposure near 1200 °C, microstructural evidence indicated that the formation and stability of a compact Cr_2_O_3_ layer on the coating surface primarily governs protection. Detailed characterisation of cold-sprayed Cr on Optimised ZIRLO™ showed that steam exposure at 1200 °C produced a continuous Cr_2_O_3_ layer that thickened to the order of several micrometres over tens of minutes (Figure 11 and Figure 12), while oxidation of the underlying Zr alloy from the coated side remained strongly suppressed within the same time window [65]. The observed deceleration of oxide growth with time is consistent with diffusion-controlled kinetics, supporting the view that the Cr_2_O_3_ layer acts as the controlling barrier during early-to-intermediate LOCA exposure times.

**Figure 13:**
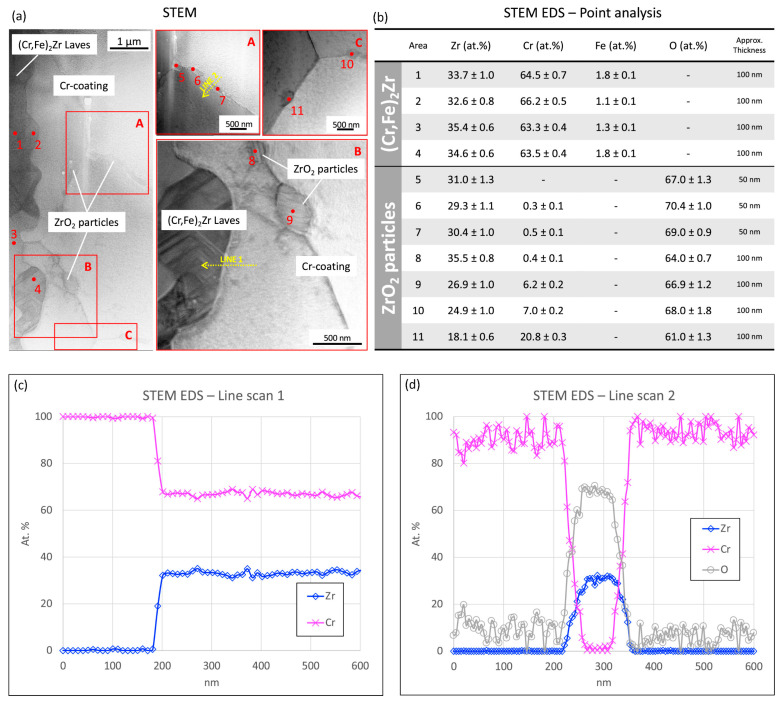
Characterisations on the cold-sprayed Cr-coated Optimised ZIRLO™ cladding exposed to simulated LOCA conditions for 40 min: (**a**) STEM images at the coating-substrate interface; (**b**) STEM-EDS point analysis results; (**c**,**d**) STEM-EDS line-scanning results [65].

At the coating/substrate interface, elevated temperature can promote chemical interaction between Cr and Zr, leading to the development of an intermetallic reaction layer (Figure 11 and Figure 12) [65]. For cold-sprayed Cr coating on Optimised ZIRLO™ cladding, a thin (micron-scale) (Cr,Fe)_2_Zr Laves-phase layer was reported after 1200 °C steam exposure. Importantly, this interfacial phase does not necessarily imply immediate degradation; instead, it may partially regulate continued interdiffusion by acting as a reaction product layer between the Cr coating and the Zr substrate. Nevertheless, high-temperature exposure also activated fast transport paths along coating grain boundaries. Zr-rich oxide particles have been observed at Cr grain boundaries near the interface after LOCA exposure, suggesting that grain-boundary processes can progressively undermine barrier performance over sufficiently long exposure times (Figure 13). A plausible longer-term degradation route is the development of a connected ZrO_2_ network along grain boundaries, which would accelerate oxygen ingress and reduce the effectiveness of the coating [65,66].

While isothermal steam oxidation tests are fundamental for elucidating the protection mechanisms of Cr coatings under LOCA-relevant temperatures, they are inherently limited to a single physical process. Predicting the full-scale accident tolerance of a fuel cladding requires understanding the complex coupling between thermomechanical deformation, burst, subsequent oxidation of exposed surfaces, and thermal shock during quenching. To address this gap, semi-integral LOCA experiments have been conducted on cold-sprayed Cr-coated claddings. These studies reveal that the coating can significantly alter the mechanical response during the temperature ramp, typically resulting in burst at higher temperatures and reduced ballooning compared to uncoated references under similar loading conditions [67,68]. However, the overall safety margin is also influenced by coating integrity in the balloon zone and by the extent to which the coating remains continuous during large hoop strain. Studies integrating ramp-to-burst, high-temperature oxidation, and quench with axial loading demonstrated that oxygen ingress through coating cracks can produce localised ZrO_2_ formation beneath crack tips, partially negating the external-surface protection otherwise provided by intact chromia [67,68]. Consequently, improvements inferred from separate oxidation tests may overestimate the benefits of coping time unless coating cracking, burst opening, and double-sided oxidation are included. In addition, earlier semi-integral work highlights that non-uniform coating thickness or local coating absence, both relevant manufacturing risks for cold spray, can limit post-quench performance, even when burst behaviour is improved [67,68].

Overall, the above literature indicates that cold-sprayed Cr coatings provide good oxidation resistance at 1200 °C steam and can delay burst and reduce ballooning. Still, their net LOCA benefit is ultimately constrained by coating uniformity, crack formation during deformation, interfacial reactions, and grain-boundary-assisted oxygen transport at longer times.

### 3.4. Further Improvement: Diffusion-Barrier Assisted Cold-Sprayed Cr Coatings

Although cold-sprayed Cr coatings are widely recognised for forming a protective Cr_2_O_3_ layer and substantially delaying Zr alloy oxidation during steam exposure at 1200 °C, their reliability can deteriorate above 1300 °C. Beyond this threshold, the Cr-Zr system can enter a regime in which the formation of Cr-Zr eutectic becomes possible, producing a liquid phase that alters the oxidation pathway and challenges the protectiveness of the Cr coating [66]. In other words, the key vulnerability at very high temperatures is not merely oxide growth, but interfacial Cr–Zr interactions leading to liquation, which can destabilise the layered structure and accelerate degradation.

To address this limitation, recent research has shifted toward bilayer architectures in which a diffusion barrier is inserted between Cr and the zirconium substrate. Yeom et al. demonstrated a fully cold-sprayed Cr–Nb system on Optimised ZIRLO™ cladding, where a thin Nb interlayer was retained between the Cr topcoat and the Zr alloy [69]. The functional role of Nb is to block Cr transport into Zr, thereby suppressing Cr–Zr eutectic melting while allowing the Cr surface to continue forming a continuous Cr_2_O_3_ layer in steam. In the 1200 °C tests, both Cr-only and Cr–Nb specimens exhibited strong oxidation resistance, but the bilayer configuration altered the interfacial reactions: instead of a Cr–Zr intermetallic zone and Cr penetration into the substrate, the system developed a thin Cr–Nb intermetallic transition layer at the Cr/Nb interface while Nb diffused into the Zr alloy without forming brittle intermetallics. This indicates that Nb can separate the oxidation-protective function of the Cr surface from the high-temperature chemical instability of the Cr–Zr couple.

The benefit of the interlayer becomes most evident at temperatures well above the Cr–Zr eutectic [69]. At 1425 °C steam exposure, Cr-only cladding showed signs consistent with rapid interdiffusion and liquid-phase involvement, accompanied by increased oxidation and pronounced dimensional instability. In contrast, the Cr–Nb-coated cladding retained coating integrity: no evidence of Cr–Zr melting was reported under the tested conditions, and the Cr topcoat remained capable of sustaining a protective Cr_2_O_3_ surface layer while Nb continued to function as a diffusion barrier. These findings support the view that introducing an interlayer is a practical route to extend cold-sprayed Cr coatings into more severe accident envelopes, specifically by preventing the interfacial liquation pathway that undermines Cr-only coatings at very high temperatures.

## 4. Other Coatings

In addition to the more extensively investigated cold-sprayed Cr-based coatings, several alternative coating systems have been explored for ATF cladding applications. Among these, FeCrAl alloy coatings and MAX phase composite coatings have attracted increasing attention due to their favourable oxidation resistance, mechanical robustness, and potential radiation tolerance. However, compared with Cr coatings, the application of cold spray to fabricate FeCrAl and MAX phase coatings remains relatively limited, with most prior studies relying on vapour phase techniques, such as PVD [70,71,72], and laser additive manufacturing [73,74]. As a result, the available literature on cold-sprayed FeCrAl and MAX phase composite coatings is comparatively sparse but nevertheless provides valuable insights into their feasibility, interfacial behaviour, and performance under accident-relevant conditions. This section, therefore, summarises and critically discusses the current state of research on these less-studied cold-sprayed coating systems, highlighting both their demonstrated advantages and the key challenges that must be addressed for their deployment in ATF cladding.

### 4.1. FeCrAl Coatings

The key mechanism that makes FeCrAl coatings a strong candidate for ATF applications is their ability to form a stable Al_2_O_3_ layer when exposed to high-temperature steam, which significantly slows oxidation [75]. This protective Al_2_O_3_ layer forms via the oxidation of Al in the FeCrAl alloy, providing a physical barrier to further oxidation. Additionally, Cr_2_O_3_ layers can form, further enhancing the corrosion resistance in steam. These properties make FeCrAl coatings highly promising for accident scenarios [51,58].

As demonstrated by Dabney et al., FeCrAl coatings significantly outperformed bare Zr alloys in oxidation tests at 1200 °C, where a protective Al_2_O_3_ layer formed on the FeCrAl surface, drastically reducing the oxidation rate compared to uncoated Zr alloy [58,76]. However, despite these promising results, a major challenge arises from the interdiffusion of Fe from the coating into the Zr alloy. This can lead to the formation of a low-melting-point eutectic phase (FeZr) at temperatures as low as 928 °C, which can undermine the structural integrity of the cladding system during high-temperature accidents [76,77].

To address this issue, a Mo interlayer has been introduced between the FeCrAl coating and the Zr alloy substrate [58]. The Mo layer can serve as a diffusion barrier, preventing Fe from diffusing into the Zr substrate and forming the problematic FeZr eutectic phase. Studies by Maier et al. and Yeom et al. have confirmed that the Mo interlayer effectively inhibited interdiffusion while allowing the FeCrAl coating to retain its oxidation-resistant properties [51,77]. This bilayer system of Mo and FeCrAl provides a robust solution for preventing eutectic formation and significantly enhances the coating’s performance under high-temperature accident conditions.

Apart from acting as a diffusion barrier, the study by Park et al. found that the Mo interlayer not only served as a diffusion barrier but also improved mechanical properties, reducing strain and preventing coating delamination during high-temperature testing [78]. Kelvin-Helmholtz instabilities were identified at the Mo/Zr interface, where vortex-like Zr bands formed, enhancing mechanical interlocking and improving the bond strength between the layers. This phenomenon occurred due to high-strain-rate deformation during cold spraying, leading to fine-scale mixing of Mo and Zr at the interface and thereby enhancing adhesion between layers.

In addition to oxidation resistance, the cold-sprayed FeCrAl coatings demonstrate superior wear resistance compared to uncoated Zr alloys. Dabney et al. evaluated the wear properties of the coatings and noted that FeCrAl coatings could mitigate grid-to-rod fretting, a common form of damage in LWRs [76]. This makes FeCrAl-coated Zr alloys a promising candidate for fuel cladding materials that must withstand both high-temperature oxidation and mechanical stresses in the reactor environment.

Furthermore, the radiation tolerance of FeCrAl-coated cladding has been assessed, with radiation tests showing that the coatings and the interfacial layers between FeCrAl and Zr alloys exhibit stable behaviour under ion irradiation [79]. The (Fe, Cr)_2_Zr intermetallic phase formed at the FeCrAl/Zr interface was found to undergo amorphisation under high-dose irradiation, without significantly degrading the thermal conductivity or mechanical properties of the coating. Moreover, the ZrC phase that forms at the interface exhibits a B1(NaCl) structure, making it highly resistant to amorphisation and radiation-induced void swelling. This structure contributes to the radiation tolerance of the coating, as the carbon vacancies in the ZrC phase enhance its defect-repair capabilities.

The combination of oxidation resistance, mechanical robustness, and radiation tolerance makes cold-sprayed FeCrAl coatings an attractive option for improving the performance of Zr-based fuel claddings in extreme accident scenarios. By incorporating a Mo interlayer, these coatings provide a viable near-term solution to the challenges posed by high-temperature transients and hydrogen generation during LOCA.

### 4.2. MAX Phase Composite Coatings

MAX phase composites are a family of layered ternary carbides and nitrides with a general chemical formula of M_n+1_AX_n_ (n = 1–3), where M is an early transition metal, A is an A-group element (typically Al or Si), and X is carbon and/or nitrogen [80]. Their crystal structure consists of alternating near-close-packed M-X octahedral layers and metallic A layers, which gives rise to a unique combination of ceramic- and metal-like properties. This hybrid nature has attracted growing interest in MAX phases as candidate materials for protective coatings in nuclear energy systems, particularly for accident-tolerant fuel cladding applications.

From a functional perspective, MAX phase composites exhibit several properties that align well with the performance requirements of ATF coatings [71]. Mechanically, they possess relatively high stiffness and hardness compared with structural metals, while retaining damage tolerance, machinability, and resistance to catastrophic brittle fracture. These characteristics are advantageous for mitigating wear, handling damage, and fretting at grid-rod contact locations on zirconium-based cladding. Thermally, MAX phase composites generally exhibit good thermal conductivity and moderate coefficients of thermal expansion, reducing thermal-mismatch stresses between the coating and the Zr-alloy substrate during both normal operation and transient heating.

Cold spray has emerged as a practical approach for depositing MAX-phase composite coatings on Zr alloys. In a pioneering study, Maier et al. successfully deposited a MAX-phase composite coating on Zircaloy-4 using low-pressure cold spraying for the first time [81]. Fine Ti_2_AlC powder feedstock (sub-20 μm) was sprayed onto the Zircaloy-4 substrate, producing coatings approximately 90–100 μm thick with relatively low porosity and no detectable phase transformation. These solid-state deposits exhibited excellent mechanical robustness: the Ti_2_AlC layer showed significantly higher hardness than the underlying Zr alloy and a markedly improved resistance to abrasive wear, which is particularly relevant to mitigating handling damage and grid-to-rod fretting. Furthermore, scratch testing demonstrated strong coating adhesion, with no spallation observed under either constant-load or progressively increasing-load conditions.

Beyond mechanical durability, Ti_2_AlC coatings have shown encouraging performance in mitigating oxidation at both moderate and accident-relevant temperatures. When exposed to air at around 700 °C, uncoated Zircaloy-4 rapidly developed an oxide layer, whereas the coated condition showed minimal oxidation at the coating/substrate boundary, suggesting that the Ti_2_AlC coating can hinder oxygen ingress into the Zr alloy. Further investigation by Maier et al. reported that the Ti_2_AlC surface formed an oxide scale (~10 μm) without oxidation at the coating/substrate interface, whereas uncoated Zr showed a thicker zirconium oxide (~25 μm), after the exposure at 1000 °C for 7 min in air [51].

However, subsequent work highlights that the long-term performance of Ti_2_AlC coatings depends strongly on interface chemistry and irradiation stability [82]. At elevated temperatures relevant to reactor operation, Al can diffuse from Ti_2_AlC into Zircaloy-4, forming Zr–Al intermetallic layers with measurable growth kinetics and a relatively high apparent activation energy for layer thickening. Because these reaction layers become part of the load-bearing and corrosion/oxidation pathway, their response to radiation damage is important. Heavy-ion irradiation experiments indicated that Ti_2_AlC experienced pronounced irradiation-induced disordering of its nanolamellar structure, while some Zr–Al intermetallic constituents could amorphise under high damage levels. Nanoindentation across the multilayer region further shows strong radiation hardening in the coating and in Zr_3_Al, whereas ZrAl_2_/ZrAl may exhibit only minor hardness changes, even when amorphisation occurs. Collectively, these studies suggest that cold-sprayed Ti_2_AlC offers a compelling combination of solid-state deposit integrity, wear resistance, and oxidation mitigation, but that practical deployment requires careful management of coating thickness, interfacial diffusion/intermetallic formation, and irradiation-driven microstructural evolution.

## 5. Industrialisation Progress

Among the cold-spray ATF candidate materials, pure Cr coating stands out for its high technological maturity and rapid industrialisation. This is mainly due to the extremely low corrosion rate in water chemistry environments, the coefficient of thermal expansion being similar to that of the Zr substrate, and the ability to quickly form a dense, self-healing C_2_O_3_ protective layer in high-temperature steam [83]. Cold-sprayed Cr coatings have progressed beyond the laboratory scale and are now being implemented on an industrial scale by international nuclear fuel suppliers. A notable example is the EnCore^®^ ATF programme by Westinghouse Electric Company. In collaboration with the University of Wisconsin-Madison and other institutions, Westinghouse has developed and optimised an industrial-scale process for applying full-length cold-spray Cr coatings on standard 4-metre-long Optimised ZIRLO™ and AXIOM^®^ zirconium alloy cladding tubes. This process includes a precision surface polishing step after cold spray deposition to ensure the coating’s surface roughness meets the requirements for reactor core hydraulic design and critical heat flux [84].

Regarding in-reactor testing, Westinghouse has inserted cold-spray Cr-coated lead test rods and assemblies into multiple commercial light-water reactors worldwide [64,85]: In the spring of 2019, the first test rods were loaded into the Byron Unit 2 nuclear power plant in the USA; In the summer of 2020, pool-side non-destructive examination data was obtained from the Doel Unit 4 nuclear power plant in Belgium; In 2023 and 2025, test assemblies containing cold-spray Cr-coated cladding and ADOPT™ doped fuel pellets with enrichments exceeding 5% were inserted into the Vogtle Unit 2 nuclear power plant to assess performance under high burnup conditions. Results from pool-side inspections at commercial reactors, as well as destructive hot cell examinations conducted at Idaho National Laboratory and Oak Ridge National Laboratory, have confirmed that after several years of high neutron irradiation and normal operating coolant flow, the cold-sprayed Cr coating remained virtually intact, with no macroscopic spallation, cracking, or fretting wear [64]. Furthermore, the cold-sprayed Cr coating effectively reduced crud deposition on the fuel rod surfaces.

FeCrAl alloys have garnered significant attention in the nuclear energy sector, primarily due to their ability to form an exceptionally stable α-Al_2_O_3_ oxide scale in high-temperature steam above 1200 °C [58,76]. The growth kinetics of this oxide film are one to two orders of magnitude slower than that of Cr_2_O_3_, providing the alloy with outstanding resistance under extreme accident conditions. Although cold spraying techniques can successfully deposit FeCrAl powder onto the surface of Zr alloys in a solid state, this technique is still in the laboratory research phase. The main challenge lies in the severe solid-state interdiffusion of Fe and Ni elements from the FeCrAl coating into the Zr alloy substrate. At temperatures as low as 928 °C, Fe and Zr can undergo a eutectic reaction, forming a liquid phase [76,77]. This interfacial melting rapidly compromises the load-bearing cross-section of the cladding, resulting in the leakage of radioactive fission products and completely negating the oxidation resistance benefits provided by the coating.

While introducing a Mo barrier layer can effectively prevent direct contact between Fe and Zr at 1200 °C [51,77], applying a multilayer structure continuously and uniformly onto full-size slender tubes, up to 4 metres long, using cold spraying presents significant engineering challenges. These challenges include the vastly different critical deformation velocities of various metal powders, the high susceptibility to interlayer debonding caused by residual stresses during multilayer spraying, the lack of scalable manufacturing processes, and the absence of long-term in-pile irradiation validation data [51,77].

Research on cold-sprayed MAX phase coatings is currently limited to the exploratory laboratory stage, with the primary challenge being the deposition mechanism of cold spraying itself [86,87]. Cold spraying relies on the severe plastic flow of particles upon high-velocity impact to form metallurgical bonds; however, MAX phase materials are inherently brittle or semi-brittle ceramics at room temperature. During the cold spraying process, MAX phase powder particles tend to fragment rather than deform plastically, resulting in extremely low deposition efficiency, poor bonding strength, and the inevitable presence of numerous microcracks and high porosity. To mitigate this issue, mechanically mixing them with ductile metal powders for co-spraying can prepare metal-based MAX phase composite coatings. However, this approach compromises the overall high-temperature tolerance of the coating.

Another critical limiting factor is the long-term stability of MAX phase materials under nuclear reactor conditions. Accelerator-based heavy ion irradiation simulation experiments suggest that when MAX phase materials are subjected to high-dose irradiation, their layered structure undergoes significant lattice disorder. Specifically, A-site elements (such as Al) are prone to being ejected and diffusing into the Zr matrix, leading to structural collapse and the formation of brittle Zr-Al intermetallic compounds [82]. This can ultimately result in coating spallation and amorphization. Given these fundamental material characteristics and deposition mechanism challenges, the application of cold-sprayed MAX phase coatings to nuclear fuel cladding still requires extensive long-term laboratory research and exploration.

## 6. Conclusions and Outlook

This review has examined the potential of cold-sprayed coatings in enhancing the performance of ATFs, particularly in improving the oxidation resistance and structural integrity of zirconium-based cladding materials used in light water reactors. Cold spray technology offers distinct advantages, most notably its ability to apply coatings at relatively low temperatures, thereby preventing oxidation and thermal degradation of sensitive materials. This capability enables the deposition of a wide array of materials, including Cr, FeCrAl alloys, and MAX phase composites, each demonstrating significant promise for improving the resilience of nuclear fuel cladding under extreme conditions.

Among cold-sprayed coating systems, Cr coatings have garnered considerable attention for their exceptional oxidation resistance and irradiation tolerance, particularly under simulated LOCA conditions. The formation of a stable Cr_2_O_3_ oxide layer upon exposure to high-temperature steam provides effective protection, significantly enhancing the cladding’s resistance to oxidation. However, challenges persist regarding the uniformity of coating thickness and the influence of interfacial reactions at elevated temperatures, which can compromise coating performance. The incorporation of diffusion-barrier-assisted Cr coatings, such as Cr-Nb bi-layer systems, has shown substantial promise in mitigating these issues. These systems effectively prevent the formation of detrimental Cr-Zr eutectics, thus enhancing the protective capacity of the coatings under severe accident scenarios.

FeCrAl coatings also offer a viable solution for ATF cladding due to their ability to form a stable Al_2_O_3_ layer, thereby significantly reducing oxidation rates in high-temperature steam environments. Despite these advantages, the interdiffusion of Fe into the Zr alloy, leading to the formation of low-melting-point FeZr eutectic phases, remains a major challenge. To address this, the introduction of Mo interlayers has proven effective in inhibiting the diffusion of Fe, thereby preserving the oxidation-resistant properties of FeCrAl coatings. The bilayer configuration, combining Mo and FeCrAl, has demonstrated improved performance in high-temperature conditions, providing a robust solution for high-temperature transients and hydrogen generation during LOCA.

MAX phase composite coatings, such as Ti_2_AlC, offer a promising alternative due to their unique combination of mechanical robustness, thermal stability, and oxidation resistance. Cold-sprayed Ti_2_AlC coatings have shown excellent wear resistance and oxidation mitigation, particularly at moderate temperatures. However, the long-term performance of these coatings is influenced by interface chemistry and irradiation stability. The formation of intermetallic layers between Ti_2_AlC and the Zr alloy, along with radiation-induced degradation of the MAX phase structure, requires careful management to ensure the longevity of the coatings in operational reactor environments.

Looking to the future, translating the promise of cold-sprayed coatings into practical reactor deployment will require a coordinated research effort across the areas of focus:

- Diffusion barrier systems: Develop and refine multi-layer architectures (e.g., Cr/Nb, FeCrAl/Mo, Cr/Ti_2_AlC) specifically designed to suppress detrimental interdiffusion and eutectic formation at the coating/substrate interface under both normal operation and accident conditions.
- Processing of brittle MAX phases: For inherently brittle ceramic phases such as MAX phases, particle fragmentation during cold spraying often results in low deposition efficiency and high porosity. Future process research should explore strategies to enhance powder deformability or optimise co-spraying approaches without compromising the high-temperature performance of the coating, ensuring uniform thickness and robust metallurgical bonding in industrially produced coatings.
- Functionally graded coatings: Investigate compositionally graded layers capable of accommodating thermal expansion mismatches and enhancing interfacial bonding, thereby reducing the risk of delamination under thermal and mechanical loading.
- Coating uniformity and defect control: Advance cold spray process parameters, including particle velocity distributions, nozzle designs, and substrate pre-heating strategies, to achieve acceptable coating thickness uniformity and minimise defects such as porosity or unbonded interfaces.
- Scale-up to full-length cladding: Address the challenges of transitioning from laboratory-scale deposition to industrial-scale, continuous and uniform coating of full-length cladding tubes. For promising composite coatings, process development must overcome challenges arising from differing critical deformation velocities among powder constituents, interlayer delamination due to residual stresses during multi-layer deposition, and the current lack of mature, scalable manufacturing processes.
- Integrated accident simulation: Move beyond separate-effects oxidation tests towards integral LOCA experiments that couple ballooning, burst, oxidation, and quenching under prototypical loading conditions. Such tests are crucial for quantifying how coating cracks and deformation affect overall safety margins.

In conclusion, cold spray technology represents a promising approach to improving the safety and performance of nuclear fuel cladding materials. Through continued advancements in coating materials, process optimisation, and long-term performance evaluation, cold-sprayed coatings can play a pivotal role in the development of accident-tolerant fuel systems, thereby contributing to the safety, sustainability, and reliability of nuclear power in the future.

## Figures and Tables

**Table 1:** Comparison of ATF cladding coatings by various deposition techniques.

Properties	Cold Spray	PVD	Laser	Plasma Spraying
Efficiency	Very high	Very low	medium	High
Porosity	Low (<1%)	Very low (<0.5%)	Low (<1%)	High (2~5%)
Oxidation	Very low	Very low	Medium	High
Bonding strength	High	High	High	Medium
Residual stress	Compressive (good for fatigue endurance)	Compressive (good for fatigue endurance)	Tensile (can cause surface cracking)	Depending on material and processing
Heat to substrate	Very low	Very low	Very high	High
Coating thickness	Flexible (10 μm~1 mm)	Very thin (<20 μm)	Thick (>100 μm)	Flexible (10 μm~1 mm)

## Data Availability

No new data were created or analysed in this study. Data sharing is not applicable.

## Abbreviations

The following abbreviations are used in this manuscript:

APT	Atom Probe Tomography
ATF	Accident-tolerant fuel
BWR	Boiling water reactor
EDS	Energy-Dispersive X-Ray Spectroscopy
FFT	Fast Fourier transform
HRTEM	High-resolution Transmission Electron Microscopy
LOCA	Loss-of-coolant accident
LWR	Light water reactor
PVD	Physical vapour deposition
PWR	Pressurised water reactor
SEM	Scanning Electron Microscopy
STEM	Scanning Transmission Electron Microscopy
TEM	Transmission Electron Microscopy
